# Evaluating the influence of straw mulching and intercropping on nitrogen uptake, crop growth, and yield performance in maize and soybean

**DOI:** 10.3389/fpls.2023.1280382

**Published:** 2023-10-13

**Authors:** Siping Liu, Lixue Wang, Liang Chang, Ismail Khan, Faisal Nadeem, Abdul Rehman, Ran Suo

**Affiliations:** ^1^ College of Water Conservancy, Shenyang Agricultural University, Shenyang, China; ^2^ School of the Environment and Safety Engineering, Jiangsu University, Zhenjiang, China; ^3^ Department of Agronomy, The University of Agriculture, DI Khan, KP, Pakistan; ^4^ Department of Agronomy, Faculty of Agriculture and Environment, The Islamia University of Bahawalpur, Bahawalpur, Pakistan; ^5^ Quality Supervision Department, Chaoyang City Water Engineering Quality and Safety Supervision Station, Chaoyang, China

**Keywords:** maize/soybean intercropping, straw mulch, crop growth, photosynthesis, N uptake, yield

## Abstract

**Introduction:**

Intercropping and straw mulching are sustainable agricultural practices that can positively affect crop growth and development, especially together.

**Methods:**

A split-plot experimental design was used to investigate the effects of intercropping and straw mulching on crop growth, crop yield, nitrogen uptake, and photosynthetic characteristics. The main plot focused on three planting patterns: soybean monoculture (S), maize monoculture (M), and maize/soybean intercropping (I). The subplot structure consisted of four levels of straw mulching (0, 4.8, 7.2, 9.6 t ha^-1^).

**Results:**

Interaction and variance analyses showed that straw mulching, intercropping, and their interaction had significant effects on plant height, stem diameter, leaf area index, chlorophyll content, nitrogen uptake, photosynthetic characteristics, and crop yield. Based on two-year averages for maize and soybean, the net photosynthetic rate (Pn) was up to 51.6% higher, stomatal conductance (Sc) was up to 44.0% higher, transpiration rate (Tr) was up to 46.6% higher, and intercellular carbon dioxide concentration (Ci) was up to 25.7% lower relative to no mulching. The maximum increases of Pn, Sc, and Tr of intercropped maize were 15.48%, 17.28%, and 23.94%, respectively, and the maximum Ci was 17.75% lower than that of monoculture maize. The maximum increase of Pn, Sc, and Tr of monoculture soybean was 24.58%, 16.90%, and 17.91%, respectively, and the maximum Ci was 13.85% lower than that of intercropped soybean. The nitrogen uptake of maize and soybean in the mulching treatment was 24.3% higher than that in the non-mulching treatment; the nitrogen uptake of intercropped maize was 34.2% higher than that of monoculture maize, and the nitrogen uptake of monoculture soybean was 15.0% higher than that of intercropped soybean. The yield of maize and soybean in the mulching treatment was 66.6% higher than that in the non-mulching treatment, the maize yield under intercropping was 15.4% higher than that under monoculture, and the yield of monoculture soybean was 9.03% higher than that of intercropped soybean.

**Discussion:**

The growth index and photosynthesis of crops are important parts of yield formation. The results of this study confirmed that straw mulching, intercropping, and their interaction can ultimately increase crop yield by improving crop growth, nitrogen uptake, and photosynthesis. This result can be used as the theoretical basis for the combined application of these measures in agriculture.

## 1 Introduction

Agricultural production is facing increasing pressure to meet growing food demand while minimizing negative environmental impacts. The gap between grain production and food demand has widened owing to the increases in population in recent decades (Godfray et al., 2010). Therefore, identification of promising and sustainable methods to produce more grain to meet food needs is urgently required. Increasing crop yield per unit area has become an inevitable objective of agricultural production. Thus, straw mulching, a resource utilization measure that can increase crop productivity (Yin et al., 2018), and intercropping, a planting mode that can increase the land equivalent ratio (Wang L. et al., 2023), have becomes critical focuses of agricultural researchers. Intercropping is a profitable strategy that efficiently uses inputs (e.g., nutrients and water), expands agricultural diversity, and increases grain yield significantly (Qin et al., 2013). Moreover, intercropping plays a crucial role in China’s grain production (Malézieux et al., 2009) and in ensuring biodiversity in a sustainable and eco-friendly manner (Lithourgidis et al., 2011). Appropriate intercropping patterns can more fully utilize agricultural resources, such as light, temperature, water, and nutrients (Hinsinger et al., 2011), and help effectively control pests and diseases. Furthermore, intercropping reduces the dependence on chemical fertilizers and pesticides in crop production, thereby lowering production costs and environmental pollution (Zhang et al., 2013).

The advantages of intercropping may depend on niche differentiation (Hector et al., 1999; Teste et al., 2014) owing to interspecific interactions (Qiao et al., 2016; Liu et al., 2022), such as nutrient complementary utilization (Li et al., 2014), and different utilization rates of light energy by different crops (Liu et al., 2017), among other factors. Under intercropping, when two crops are planted together, the competition and beneficial effects between adjacent plants will affect the growth of crops (Teste et al., 2014; Zhang et al., 2014). Positive interactions between crops increase the balance among cropping systems and enhance the economic and ecological benefits (Li et al., 2007; Ahmad et al., 2019; Hong et al., 2023). Under China’s limited cultivated land resources, intercropping is a suitable method to improve land resource utilization efficiency and attain higher crop yield (Li R. et al., 2020). Therefore, intercropping of the cereal maize (*Zea mays* L.) with the legume soybean (*Glycine max* L.) offers multiple advantages, such as high land productivity, improved resource use, and decreased disease incidence, ultimately leading to higher grain yield (Iqbal et al., 2019; Chang et al., 2020). This intercropping system is enabled by the growing seasons and growth periods of maize and soybean being the same, which permits them to be harvested simultaneously, reducing the number of field operations and thereby improving production efficiency (Liu et al., 2022). When grown together, maize and soybean have a long period of overlap, a high rate of light capture, and a large photosynthetic area that can sustainably improve the light energy utilization rate of the system (Xia et al., 2013).

Straw mulching, another sustainable method of increasing agricultural effificiency, is a way of straw returning to the fifield, and it includes whole straw mulching, deep plowing, and broken mixed mulching (Khan et al., 2022a). Studies have shown that straw mulching can increase the content of organic carbon and nutrients in topsoil, improve the stability of soil aggregates (Kumar et al., 2012; Zuber et al., 2015), improve soil temperature, and enhance the suitability of the environment for crop growth (Khan et al., 2022b). Straw mulching has positive effects, including reducing soil water evaporation, increasing soil water retention and crop yield, and high-water use efficiency. It is also a widely promoted cultivation technique in arid and semi-arid areas (Liy et al., 2021). Crop residues maintain soil temperature balance, lower the maximum soil temperature, and increase soil microbial biomass and enzymatic activities (Masciandaro et al., 2004; Chen et al., 2007; Guo et al., 2013). Straw mulch application in maize cultivation decreases water evapotranspiration and soil water consumption and increases water use efficiency, resulting in higher yield and overall economic benefit (Wang L. et al., 2023). Plants perform a variety of essential functions that are critical to their survival and growth. These functions include photosynthesis, gas exchange, water transpiration, and the production of nutrients, all of which take place primarily in the leaves (Bucher et al., 2021). The crop grain yield and dry matter is derived mainly from the assimilation products of photosynthesis that occur in plant leaves (Makino & Amane, 2021), and an increase of total leaf area will prolong the functional period of leaves, which is beneficial to the accumulation of photosynthetic products and ultimately affects crop yield (Zhang et al., 2014).

The present study investigates the effects of straw mulching and intercropping on the leaf traits, physiological characteristics, and yield of maize and soybean in Northeast China. While previous studies have examined these agronomic methods separately (Chen et al., 2007; Ahmed et al., 2018), this study focuses on the combined effects of straw mulching and intercropping on agricultural production systems. Given the close relationship between leaf traits, physiological characteristics, and crop yield, this study explores the impact of different straw mulching levels on leaf area index, plant nitrogen uptake, chlorophyll content, leaf photosynthetic characteristics, and crop yield under intercropping. The objective is to determine the optimal straw mulching amount and planting pattern to maximize yield and photosynthetic performance under combined straw mulching and intercropping. Through this investigation, the study seeks to provide technical and theoretical support for the effective combination of intercropping and straw mulching in China’s agricultural production and contribute to developing the effectiveness of these practices. Ultimately, the findings of this study are intended to enhance crop productivity and grain yield in China.

## 2 Materials and methods

### 2.1 Experimental site

This experiment was carried out at the comprehensive experiment base of the College of Water Conservancy of Shenyang Agricultural University (123.57°E, 41.83°N, average altitude 44.7 m) from May to September in 2017 and 2018. The experiment base is located in the eastern part of Shenyang, which has a temperate continental monsoon climate, and the average annual rainfall is 703.4 mm. During the growth period of soybean and maize crops (May to September), the precipitation was relatively concentrated. In 2017, the precipitation during the growth period was 420.5 mm, and the total annual rainfall was 463.8 mm. In 2018, the precipitation during the growth period was 594.6 mm, and the total annual rainfall was 665.9 mm. The soil in the test area is tidal brown soil.

The key nutrient contents of the soil were as follows: total nitrogen 1.24 g kg^-1^, available phosphorus 62.4 mg kg^-1^, total potassium content 130.2 mg kg^-1^, and organic matter content 33.9 g kg^-1^. The pH was 7.14, and the electrical conductivity (EC) was 1.38 mS cm^-1^. The soil bulk density of the 20-cm soil layer was 1.36 g cm^-3^, and that of the 40-cm soil layer was 1.41 g cm^-3^. The soil was evenly distributed and was a typical representative soil in this area. The field water holding rate was 30.28%, and the wilting coefficient was 18%.

### 2.2 Experimental design

In this experiment, a split-plot experimental design was used across three planting patterns, which were soybean monoculture (S), maize monoculture (M), and maize/soybean intercropping (I). Thesubplots had four different levels of straw mulching: 0 t ha^-1^ (M0), 4.8 t ha^-1^ (M1), 7.2 t ha^-1^ (M2), 9.6 t ha^-1^ (M3). Thus there were a total of 12 treatments, as presented in **Table 1**. Each treatment was set up with three replicates, for a total of 36 test plots; each test plot area was 18 m^2^ (3 m × 6 m). The maize variety planted in the experiment was Dongdan 80, and the soybean variety was Dongdou 1, which both have high seed yield and great yield potential and are widely used in this area, making them particularly suitable for this study. The intercropping treatment of maize and soybean involved their seeds being planted at a ratio of 1:1, and the row spacing of maize and soybean was 0.4 m. For monoculture maize, the row spacing was 0.4 m, and the plant spacing was 0.3 m. For monoculture soybean, the row spacing was 0.4 m, and the hole spacing was 0.2 m, with two plants per hole. No additional irrigation was applied during the experiment. The only supplementary water source for the crops came from rainfall. The mulching maize straw of the previous season’s crop was cut into 2- to 3-cm pieces and kept in dark and ventilated storage. During sowing, this mulching straw was shallowly buried in the 0–15 cm plough layer soil. The growth period of maize and soybean were basically the same. Soybean and maize were sown at the same time on both May 8, 2017 and May 5, 2018.

**Table 1:** Experimental Treatment numbers.

Straw mulching level (t·ha^-1^)		Planting pattern		Treatment numbers
0	M0	Soybean monoculture	S	M0S
4.8	M1			M1S
7.2	M2			M2S
9.6	M3			M3S
0	M0	Maize monoculture	M	M0M
4.8	M1			M1M
7.2	M2			M2M
9.6	M3			M3M
0	M0	Maize/soybean intercropping	I	M0I
4.8	M1			M1I
7.2	M2			M2I
9.6	M3			M3I

The amount of fertilizer applied was based on the specific intercropping treatment. The amount of fertilizer used in intercropping should be limited to the amount of nitrogen required by soybeans, not more than 60 kg ha^-1^ pure nitrogen (Central Agricultural Broadcasting School, 2022). Therefore, each treatment involved the application of compound fertilizer (N-P_2_O_5_-K_2_O contents of 13%, 17%, and 15%, respectively) as base fertilizer at one-time according to the standard application of 450 kg ha^-1^. No additional fertilizer was applied during each growth stage, and unified management measures were adopted for field crops.

### 2.3 Experimental data determination

#### 2.3.1 Rainfall and monthly mean temperature

**Figure 1:**
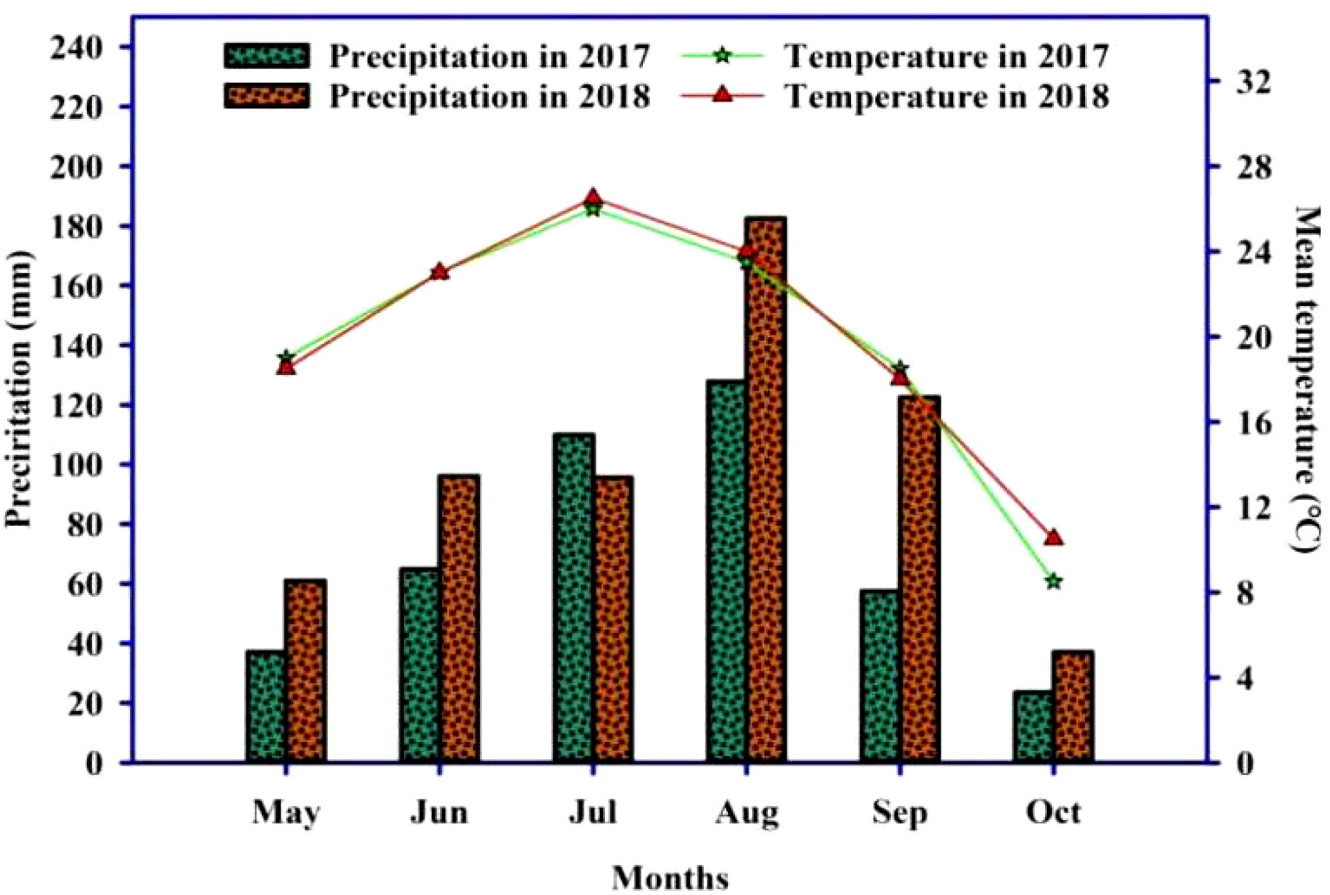
Rainfall and monthly mean temperature during maize and soybean growth period (May to October) in 2017 and 2018.

Precipitation and temperature data were obtained from the meteorological data observed by the Dongling hydrological station, which is 3.8 km from the experiment site. The rainfall and temperature data during the growth period of maize and soybean (May–October) are shown in **Figure 1**.

#### 2.3.2 Crop growth dynamics

Three maize and soybean plants with uniform growth were selected for each plot. The height from the ground to the top of each plant was measured, and the plants were chosen to be labeled and observed every ten days. The average stem diameter was measured with a vernier caliper at each growth stage. The measurement site was the third internode of the stem base from the bottom of the plant. The plants were marked and observed every ten days.

#### 2.3.3 Leaf area index

Three maize and soybean plants with consistent growth were selected during each growth stage. A ruler was used to measure the length and width of maize ear leaves and soybean leaves, and the width was measured at the widest part of the leaf. The leaf area per unit area in the plot was calculated, and the single leaf area and leaf area index (LAI) were calculated using equations (1) and (2).


(1) $$S_{n}=\frac{L_{n}\times W_{n}\times 0.75}{10000}$$

where *S_n_* is single leaf area, in m^2^; *l_n_* and *W_n_* are the length and width of a single leaf, in cm.


(2) $$LAI=\frac{S•n}{S_{L}},$$

where *S* is leaf area per plant; *N* is the number of plants in the plot area; S_L_ is the plot land area, in m^2^.

#### 2.3.4 Chlorophyll content

The leaf chlorophyll content of crops was measured by a CCM-300 chlorophyll content measuring instrument (Opti-Sciences, Inc., Hudson, NH, USA). Three representative plants were selected in each maize and soybean monoculture plot, and three maize and three soybean plants were selected in the intercropping plot. The measurement sites were fully expanded functional leaves in the middle and upper parts of each selected plant. The average value was recorded, and the measurement time was the growth period of the crop.

#### 2.3.5 Determination of photosynthetic parameters

In each growth stage, three maize and soybean plants with uniform growth were selected in each treatment, and the photosynthetic parameters of maize and soybean plants were measured. The photosynthetic parameters, including net photosynthetic rate (Pn), stomatal conductance (Sc), intercellular carbon dioxide concentration (Ci), and transpiration rate (Tr), were measured using the LI-6400 (LI-COR Company, Lincoln, NE, USA) portable photosynthetic instrument. To avoid any potential edge effect, in the intercropping treatment, plants were selection from among the middle row of plants for measurement. The measurement site was the ear leaf of maize plants and the third leaf of soybean plants, which were fully expanded functional leaves in the middle and upper parts of the crops, and measurements were recorded from 9:00 to 12:00 a.m. during sunny weather.

#### 2.3.6 Crop nitrogen uptake

The crop samples for each period were collected, and a grinderfilled with maize and soybean stems, leaves, and grains was used to crush the dried plant samples. After samples were thoroughly mixed, they were transferred into a plastic sealed bag for the measurement of crop nitrogen uptake, and the N nutrient content of the plant was measured using a Kjeldahl nitrogen analyzer (KDN-520; Sayas Technology Co., Ltd., Jilin, China).

#### 2.3.7 Yield and yield components

The two crops were harvested manually when maize and soybean had matured, and crop samples were collected from a random 2 × 2 m area for threshing and yield measurement in the monoculture area. To avoid any potential edge effect of the plot, maize and soybean crop samples were collected from the inner rows with a length of more than 2 m in the intercropping plot, and maize and soybean plants were selected for threshing and yield measurement. The grain moisture content of maize and soybean was measured using a moisture meter. The dry weight was measured after artificial threshing, and the yield per unit area was calculated. The yield of maize was finally converted to the grain yield based on 14% moisture content (dry weight), and the soybean yield was transformed based on a moisture content of 13% (dry weight). The calculation formula is dry weight = original weight × (1 - original moisture content %)/(1 - moisture content after drying %), and the yield per unit area is expressed as t ha^-1^.

Five maize and soybean plants with consistent development were selected from each plot to measure the yield traits, including ear length, ear diameter, grain number per ear, and 100-grain weight, of maize at the end of the growing season. The yield traits, including pod number per plant, grain number per plant, grain weight per plant, and 100-grain weight, of soybean plants were also determined.

### 2.4 Data analysis

The data for all collected parameters were processed using Excel 2021 (Microsoft Corp., Redmond, WA, USA) and analyzed by analysis of variance (ANOVA) using the statistical software package SPSS 24 (IBM Corp., Armonk, NY, USA). To test differences among groups based on both growing seasons, the *post-hoc* least significant difference (LSD) test was applied at the *P*< 0.05 probability level. SigmaPlot (Ver.12.5, Systat Software Inc., Palo Alto, CA, USA) was used for graphing.

## 3 Results

### 3.1 Effects of straw mulching and intercropping on plant height and stem diameter of crops

**Figure 2:**
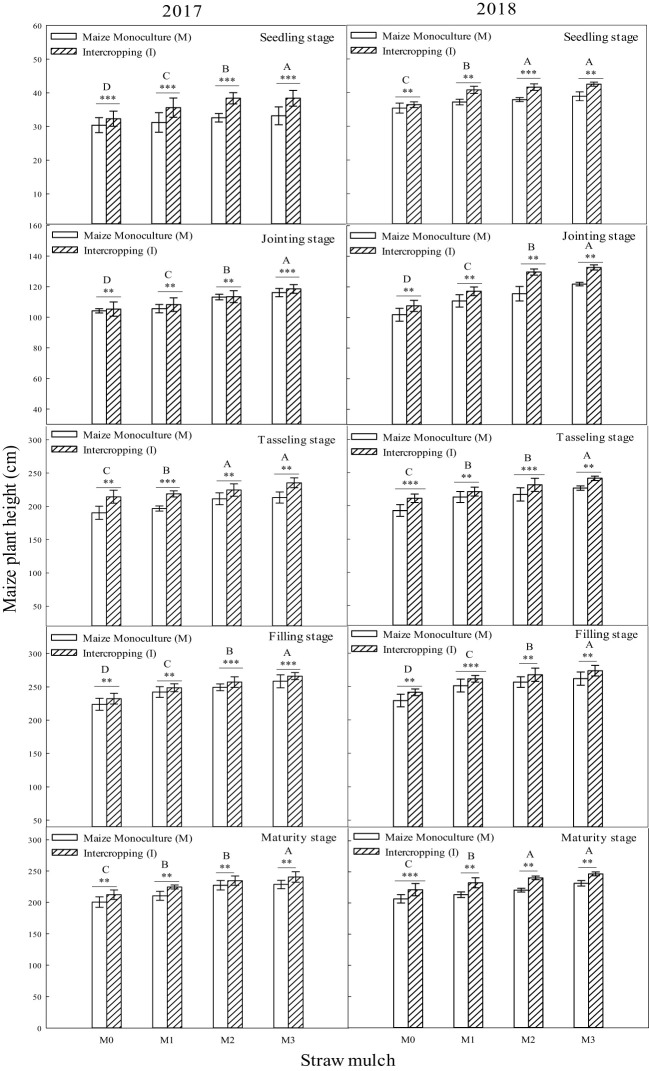
Effect of planting pattern and straw mulch on maize plant height. Values labelled with different capital letters indicate significant differences between straw mulch treatments (*p*< 0.05). Straw mulching levels: 0 t ha^-1^ (M0), 4.8 t ha^-1^ (M1), 7.2 t ha^-1^ (M2), 9.6 t ha^-1^ (M3). ** and *** indicate significant differences at the *P* < 0.01, and *P* < 0.001 levels, respectively.

The plant height of maize reached its maximum at the filling stage, and the effect of the combined treatment decreased slightly compared with the filling stage from one year to the next (**Figure 2**). The interaction between straw mulching and planting pattern significantly affected maize plant height at each growth stage (**Table 2**).

**Table 2:** Variance analysis of planting pattern and straw mulch on maize plant height.

Year	Source of variation	Seedling stage	Jointing stage	Tasseling stage	Filling stage	Maturity stage
2017	P	315***	121**	52**	254**	134**
	M	246***	98**	122**	222**	25**
	P×M	14.6**	125.2*	84.3**	26.4**	26.4**
2018	P	114***	56**	26**	112**	141**
	M	86***	38**	67**	64**	36**
	P×M	137**	235**	5.32**	10.6**	22.4**

The values in the table represent the F-values of the main effect and interaction term in an analysis of variance; ns means not significant; *, * *, and * * * indicate significant differences at the P< 0.05, P< 0.01, and P< 0.001 levels, respectively; P, planting pattern; M, straw mulch.

Under the same mulching level, the plant height of intercropped and monoculture maize was significantly different (*P*< 0.05). Under the M0, M1, M2, and M3 mulching levels, the plant height of intercropped maize was 6.57%, 7.76%, 9.38%, and 14.3% higher than that of monoculture maize (based on a two-year average during the whole growing season, as indicated for the results presented below unless otherwise noted), indicating that intercropping had a significant effect on maize plant height (**Figure 2**). The plant height of maize under M1, M2, and M3 levels was significantly higher than under M0 (*P*< 0.05). Under monoculture conditions, the plant height of M1, M2, and M3 increased by 6.29%, 10.6%, and 13.2%, respectively, compared with that of M0. Under intercropping, maize plant height increased by 7.48%, 13.6%, and 21.4%, respectively (**Figure 2**).

**Figure 3:**
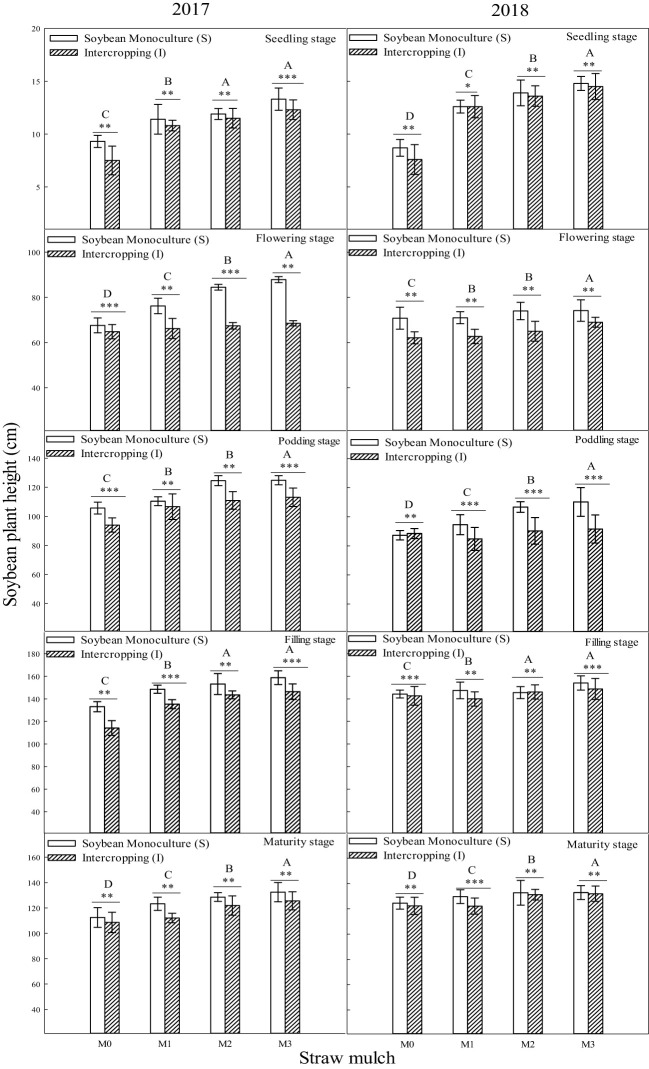
Effect of planting pattern and straw mulching on soybean plant height. Values labeled with different letters indicate significant differences between straw mulch treatments (*P<* 0.05). Straw mulching levels: 0 t ha^-1^ (M0), 4.8 t ha^-1^ (M1), 7.2 t ha^-1^ (M2), 9.6 t ha^-1^ (M3); *, ** and *** indicate significant differences at the *P* < 0.05, *P* < 0.01, and *P* < 0.001 levels, respectively.

Soybean plant height reached its maximum at the filling stage, andheight at maturity decreased slightly compared with the filling stage during the two years (**Figure 3**). The interaction between straw mulching and planting patterns significantly affected soybean plant height at each growth stage (**Table 3**).

**Table 3:** Variance analysis of straw mulch and planting pattern on soybean plant height.

Year	Source of variation	Seedling stage	Flowering stage	Podding stage	Filling stage	Maturity stage
2017	P	78**	64**	65**	31*	35**
	M	113**	121**	19*	95**	62**
	P×M	15.2**	25.5**	24.3**	16.6**	26.4**
2018	P	56**	133**	224**	112**	99**
	M	8.9**	214**	366**	98**	76**
	P×M	11.5**	12.3**	15.2*	5.68*	32.6**

The values in the table represent the F-value of the interaction term in an analysis of variance; ns means not significant; *, * *, and * * * indicate significant differences at the P< 0.05, P< 0.01, and P< 0.001 levels, respectively; P, planting pattern; M, straw mulch.

Under the same mulching level, the plant height of intercropped and monoculture soybean was significantly different (*P*< 0.05). Under the M0, M1, M2, and M3 mulching levels, the plant height of intercropped soybean decreased by 7.48%, 8.22%, 10.1%, and 17.1%, respectively, indicating that intercropping inhibited soybean plant height (**Figure 3**). As shown in **Figure 3**, the soybean plant height under M1, M2, and M3 was significantly (*P*< 0.05) higher than that under M0. Under monoculture conditions, the soybean plant height under M1, M2, and M3 increased by 6.94%, 14.7%, and 25.8%, respectively, compared with M0. Under intercropping, soybean plant height increased by 6.20%, 12.1%, and 15.5%,respectively (**Figure 3**).

**Figure 4:**
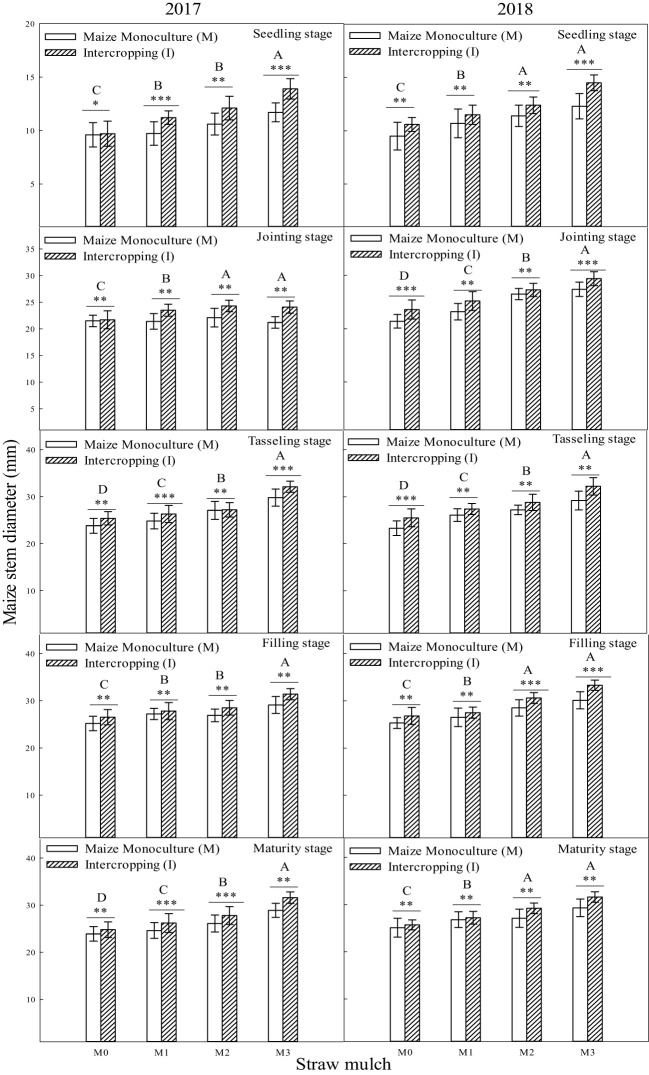
Effect of straw mulch and planting pattern on stem diameter of maize. Bars labeled with different capital letters indicate significant differences between straw mulch treatments (*P<* 0.05). Straw mulching levels: 0 t ha^-1^ (M0), 4.8 t ha^-1^ (M1), 7.2 t ha^-1^ (M2), 9.6 t ha^-1^ (M3). *, **, and *** indicate significant differences at the *P<* 0.05, *P*< 0.01, and *P*< 0.001 levels, respectively.

The stem diameter of maize reachedits maximum at the filling stage, and the diameter at maturity decreased slightly compared with the fillingstage during the two years (**Figure 4**). Except for the seedling stage in 2018, the interaction between straw mulching and planting patterns significantly affected the stem diameter of maize at each growth stage (**Table 4**).

**Table 4:** Variance analysis of straw mulch and planting pattern on stem diameter of maize.

Year	Source of variation	Seedling stage	Jointing stage	Tasseling stage	Filling stage	Maturity stage
2017	P	115**	253**	241**	67**	200**
	M	10*	178**	312**	86**	142**
	P×M	11.2**	13.7**	7.26*	6.18*	8.35*
2018	P	35**	266**	25**	122**	162**
	M	96**	232**	48**	97**	213**
	P×M	0.27ns	1044***	12.4**	6.8*	14.2**

The values in the table represent the F-values of the interaction term in an analysis of variance; ns means not significant; *, * *, and * * * indicate significant differences at the P< 0.05, P< 0.01, and P< 0.001 levels, respectively; P, planting pattern; M, straw mulch.

Under the same mulching level, maize’s stem diameter significantly differed between intercropping and monoculture (*P<*0.05). Under the M0, M1, M2, and M3 mulching levels, the stem diameter of intercropped maize was 6.31%, 8.78%, 10.9%, and 11.7% higher than that of monoculture maize, respectively. The two-year average shows that intercropping significantly increases maize stem diameter (**Figure 4**). It can also be seen from the figure that the stem diameter of M1, M2, and M3 was significantly (*P<* 0.05) higher than that of M0. Under monoculture conditions, the stem diameter of maize in M1, M2, and M3 increased by 3.86%, 9.18%, and 15.9%, respectively, compared with M0. Under intercropping, stem diameter of maize increased by 6.28%, 13.9%, and 21.7%, respectively (**Figure 4**).

**Figure 5:**
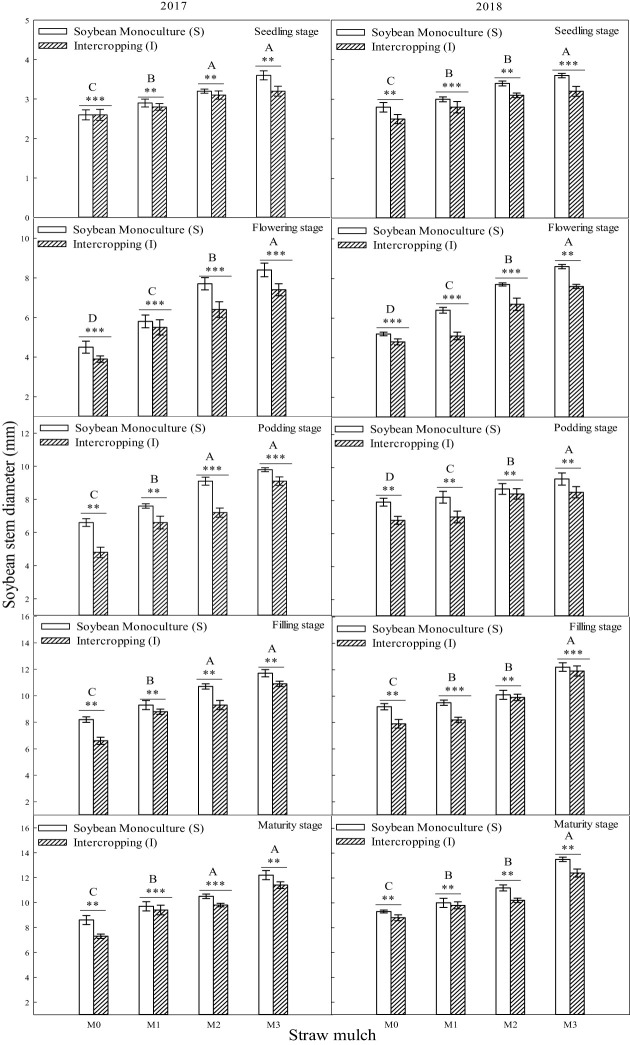
Effect of straw mulch and planting pattern on stem diameter of soybean. Bars labeled with different capital letters indicate significant differences between straw mulch treatments (*P<* 0.05). Straw mulching levels: 0 t ha^-1^ (M0), 4.8 t ha^-1^ (M1), 7.2 t ha^-1^ (M2), 9.6 t ha^-1^ (M3). ** and *** indicate significant differences at the *P* < 0.01, and *P* < 0.001 levels, respectively.

The stem diameter of soybean reachedits maximum at the filling stage, anddiameter at maturity decreased slightly compared with the filling stage during the two years (**Figure 5**). In addition to the seedling stage in 2018, the interaction between straw mulching and planting patterns significantly affected soybean stem diameter at each growth stage (**Table 5**).

**Table 5:** Variance analysis of straw mulch and planting pattern on steam diameter of soybean.

Year	Source of variation	Seedling stage	Flowering stage	Podding stage	Filling stage	Maturity stage
2017	P	108**	332***	86**	122**	35**
	M	34**	278***	154**	57**	61**
	P×M	12.3**	8.77**	5.69*	5.22*	9.28**
2018	P	132**	52	95**	276**	43**
	M	69**	168	37**	164**	251**
	P×M	0.46ns	351**	2.31*	4.6*	13.2**

The values in the table represent the F-values of the interaction term in an analysis of variance; ns means not significant; *, * *, and * * * indicate significant differences at the P< 0.05, P< 0.01, and P< 0.001 levels, respectively; P, planting pattern; M, straw mulch.

The intercropped and monoculture soybean plant height significantly differed under the same mulching level (*P<* 0.05). Similar to the plant height of soybean, the stem diameter of monoculture soybean increased by 3.19%, 6.31%, 11.4%, and 12.3%, respectively, under M0, M1, M2, and M3 mulching levels, indicating that intercropping inhibited the soybean stem diameter (**Figure 5**). As also seen in **Figure 5**, the soybean stem diameter of M1, M2, and M3 was significantly (*P<* 0.05) higher than that of M0. Under monoculture conditions, the stem diameter of M1, M2, and M3 increased by 9.60%, 27.4%, and 46.1%, respectively, compared with M0. Under intercropping conditionssoybean stem diameter increased in these mulching treatments by 6.39%, 18.1%, and 34.4%, respectively, based on the two-year average (**Figure 5**).

### 3.2 Effects of straw mulching and planting patterns on crop LAI

The changes in LAI during the growth period of maize under different planting patterns and straw mulching levels are shown in **Table 6**; straw mulching amount, planting pattern, and their interaction significantly affected maize LAI (*P*< 0.05). The LAI of maize was significantly affected by the amount of straw mulching at each growth stage, with higher mulching levels consistently associated with higher LAI (**Table 6**). The LAI of maize increased significantly under intercropping. The LAI of maize increased by 5.73%, 5.99%, 7.95%, and 10.5%across the four straw mulching levels from M0 to M3, respectively, throughout the whole growth periodbased on two-year averages. The increasein LAI of intercropped maize was the greatest compared with that of monoculture maize under M3 treatment.

**Table 6:** Effect of planting pattern and straw mulch treatments on maize leaf area index (LAI).

Growth stage	Treatment	2017			2018		
		Monoculture	Intercropping	Mean	Monoculture	Intercropping	Mean
Seedling	M0	0.49 ± 0.06b	0.51 ± 0.04a	0.50D	0.52 ± 0.07b	0.53 ± 0.08a	0.53D
	M1	0.53 ± 0.03b	0.54 ± 0.06a	0.54C	0.56 ± 0.03b	0.60 ± 0.08a	0.58C
	M2	0.56 ± 0.04b	0.58 ± 0.12a	0.57B	0.59 ± 0.04b	0.62 ± 0.04a	0.61B
	M3	0.63 ± 0.02b	0.65 ± 0.07a	0.64A	0.63 ± 0.07b	0.65 ± 0.01a	0.64A
Jointing	M0	0.93 ± 0.09b	1.18 ± 0.16a	1.06D	1.13 ± 0.13b	1.27 ± 0.08a	1.20C
	M1	1.13 ± 0.32b	1.63 ± 0.04a	1.38C	1.93 ± 0.18b	1.52 ± 0.33a	1.73B
	M2	1.38 ± 0.16b	1.82 ± 0.06a	1.60B	1.87 ± 0.12b	1.71 ± 0.23a	1.79B
	M3	1.75 ± 0.08b	1.86 ± 0.10a	1.81A	2.11 ± 0.13b	1.97 ± 0.17a	2.04A
Tasseling	M0	4.61 ± 0.13b	4.83 ± 0.08a	4.72D	4.43 ± 0.03b	4.59 ± 0.13a	4.51D
	M1	4.76 ± 0.24b	5.22 ± 0.21a	4.99C	4.92 ± 0.28b	5.29 ± 0.18a	5.11C
	M2	5.12 ± 0.15b	5.52 ± 0.24a	5.32B	5.00 ± 0.18b	5.48 ± 0.27a	5.24B
	M3	5.39 ± 0.38b	6.09 ± 0.16a	5.74A	5.69 ± 0.54b	6.38 ± 0.13a	6.04A
Filling	M0	4.15 ± 0.19b	4.30 ± 0.08a	4.23C	4.09 ± 0.40b	4.18 ± 0.11a	4.14D
	M1	4.48 ± 0.28b	4.55 ± 0.35a	4.52B	4.47 ± 0.31b	4.68 ± 0.31a	4.58C
	M2	4.60 ± 0.13b	4.81 ± 0.12a	4.71A	4.77 ± 0.23b	4.87 ± 0.10a	4.82B
	M3	4.61 ± 0.22b	5.32 ± 0.33a	4.97A	5.15 ± 0.20b	5.61 ± 0.25a	5.38A
Maturity	M0	2.43 ± 0.21b	2.68 ± 0.08a	2.56D	2.53 ± 0.12b	2.69 ± 0.04a	2.61C
	M1	2.75 ± 0.03b	2.9 ± 0.05a	2.83C	2.83 ± 0.12b	3.13 ± 0.05a	2.98C
	M2	3.18 ± 0.29b	3.42 ± 0.05a	3.30B	3.12 ± 0.18b	3.76 ± 0.17a	3.44B
	M3	3.37 ± 0.09b	3.86 ± 0.08a	3.62A	3.80 ± 0.26b	4.21 ± 0.06a	4.01A
ANOVA	P	329.8^**^	244.8^**^		58.8^**^	122^**^	
	M	422.3^**^	115.8^**^		124^**^	16.8^**^	
	P×M	38.8^**^	24.3^**^		575.3^**^	174.5^**^	

Values labeled with different capital letters indicate significant differences in maize LAI between different amounts of straw mulching (P< 0.05), while different lowercase letters indicate significant differences in maize LAI between intercropping and monoculture (P< 0.05). Straw mulching levels: 0 t ha^-1^ (M0), 4.8 t ha^-1^ (M1), 7.2 t ha^-1^ (M2), 9.6 t ha^-1^ (M3). * * indicate significant differences at the P< 0.01 level.

As shown in **Table 7**, straw mulching amount, planting pattern, and their interaction significantly affected soybean LAI (*P<* 0.05). The LAI of soybean was significantly affected by the amount of straw mulching at each growth stage, with LAI consistently increasing with mulching amount. The soybean LAIunder mulching treatments M1, M2, and M3 were 10.8%, 21.8%, and 38.7% higher than that under non-mulching treatment M0, respectively, under intercroppingbased on the two-year averagethroughout the whole growth period; the LAI of soybean under mulching treatments M1, M2, and M3 was 11.7%, 23.2% and 41.6% higher than M0 in monoculture, respectively. The planting pattern significantly affected soybean LAI (*P<* 0.05). Under the four straw mulching levels from M0 to M3, the LAI of monoculture soybean was 6.00%, 6.87%, 7.21%, and 8.24% higher than that of intercropped soybean, respectively. The increase of LAI in intercropped soybean relative to monoculture soybean was the largest under M3 treatment.

**Table 7:** Effect of planting pattern and straw mulch treatments on soybean leaf area index (LAI).

Growth stage	Treatment	2017			2018		
		Monoculture	Intercropping	Mean	Monoculture	Intercropping	Mean
Seedling	M0	0.48 ± 0.09a	0.46 ± 0.11b	0.47C	0.52 ± 0.07a	0.48 ± 0.08b	0.50D
	M1	0.55 ± 0.13a	0.50 ± 0.05b	0.53C	0.58 ± 0.06a	0.53 ± 0.08b	0.56C
	M2	0.60 ± 0.01a	0.56 ± 0.12b	0.58B	0.64 ± 0.05a	0.58 ± 0.07b	0.61B
	M3	0.72 ± 0.03a	0.62 ± 0.06b	0.67A	0.78 ± 0.04a	0.65 ± 0.09b	0.72A
Flowering	M0	0.72 ± 0.22a	0.70 ± 0.02b	0.71D	0.82 ± 0.15a	0.78 ± 0.12b	0.80D
	M1	0.81 ± 0.42a	0.78 ± 0.04b	0.80C	0.98 ± 0.22a	0.88 ± 0.29b	0.93C
	M2	0.89 ± 0.33a	0.84 ± 0.23b	0.87B	1.06 ± 0.14a	0.97 ± 0.19b	1.02B
	M3	0.97 ± 0.19a	0.94 ± 0.21b	0.96A	1.15 ± 0.16a	1.07 ± 0.15b	1.11A
Podding	M0	0.88 ± 0.21a	0.80 ± 0.18b	0.84D	0.91 ± 0.07a	0.88 ± 0.14b	0.90D
	M1	0.93 ± 0.28a	0.91 ± 0.15b	0.92C	0.99 ± 0.05a	0.93 ± 0.18b	0.96C
	M2	1.02 ± 0.28a	0.99 ± 0.18b	1.01B	1.16 ± 0.05a	1.08 ± 0.25b	1.12B
	M3	1.34 ± 0.23a	1.23 ± 0.15b	1.29A	1.38 ± 0.21a	1.29 ± 0.17b	1.34A
Filling	M0	0.80 ± 0.18a	0.75 ± 0.11b	0.78D	0.92 ± 0.15a	0.88 ± 0.05b	0.90C
	M1	0.85 ± 0.28a	0.80 ± 0.22b	0.83C	0.99 ± 0.18a	0.96 ± 0.36b	0.98B
	M2	0.90 ± 0.18a	0.88 ± 0.25b	0.89B	1.11 ± 0.29a	1.00 ± 0.16b	1.06A
	M3	1.00 ± 0.23a	0.96 ± 0.28b	0.98A	1.18 ± 0.16a	1.11 ± 0.52b	1.15A
Maturity	M0	0.56 ± 0.17a	0.52 ± 0.09b	0.54C	0.63 ± 0.18a	0.58 ± 0.37b	0.61D
	M1	0.69 ± 0.31a	0.61 ± 0.27b	0.65B	0.72 ± 0.19a	0.67 ± 0.28b	0.70C
	M2	0.75 ± 0.11a	0.69 ± 0.15b	0.72B	0.79 ± 0.15a	0.73 ± 0.19b	0.76B
	M3	0.85 ± 0.18a	0.78 ± 0.28b	0.82A	0.88 ± 0.26a	0.82 ± 0.18b	0.85A
ANOVA	P	228.01^**^	421.35^**^		526.8^**^	172.36^**^	
	M	1132.42^**^	1243.2^**^		1128.3^**^	520.65^**^	
	P×M	35.96^**^	543.9^**^		135.64^**^	70.25^**^	

Values labeled with different capital letters indicate significant differences in soybean LAI between different amounts of straw mulching (P< 0.05), while different lowercase letters indicate significant differences in soybean LAI between intercropping and monoculture (P< 0.05). Straw mulching levels: 0 t ha^-1^ (M0), 4.8 t ha^-1^ (M1), 7.2 t ha^-1^ (M2), 9.6 t ha^-1^ (M3). * * indicate significant differences at the P < 0.01 level.

### 3.3 Effects of straw mulching and planting patterns on crop chlorophyll content

**Figure 6:**
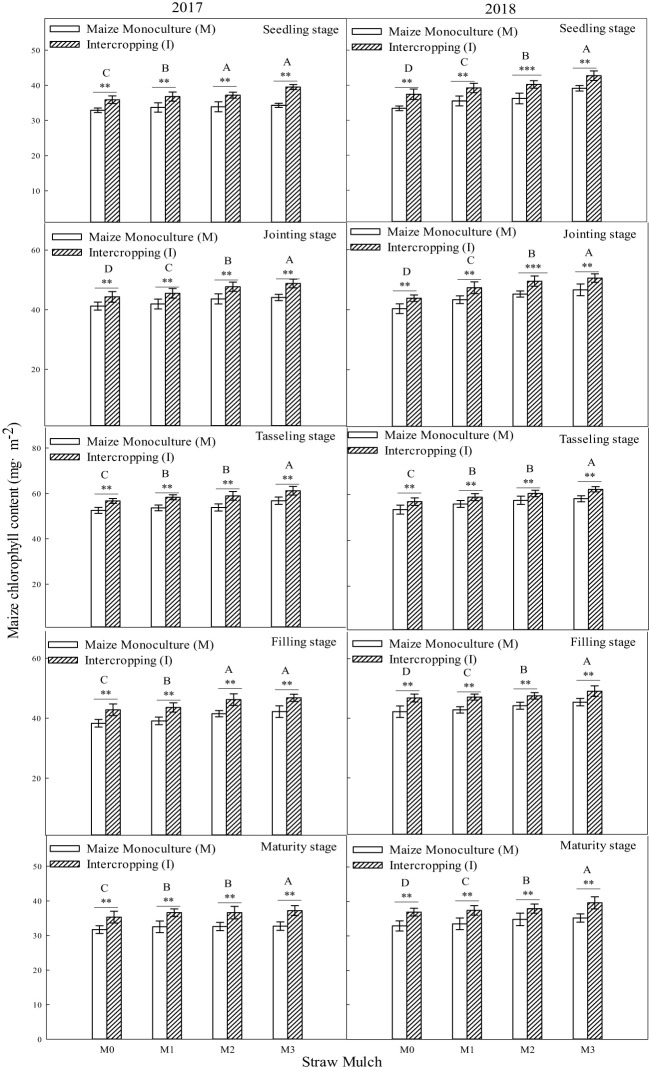
Effects of straw mulching and planting patterns on chlorophyll content of maize at different growth stages. Bars labeled with different capital letters indicate significant differences between straw mulch treatments (*P<* 0.05). Straw mulching levels: 0 t ha^-1^ (M0), 4.8 t ha^-1^ (M1), 7.2 t ha^-1^ (M2), 9.6 t ha^-1^ (M3). ** and *** indicate significant differences at the *P* < 0.01, and *P* < 0.001 levels, respectively.

It can be seen from **Figure 6** that the chlorophyll content during the growth period of maize was significantly affected by straw mulching and planting pattern (*P<* 0.05). As mulching level increased from M0 to M3, chlorophyll content of maize also increased. Under intercropping, the chlorophyll content of maize under mulching treatments M1 to M3 was 3.18%, 6.27%, and 9.59% higher thanM0, respectively. The chlorophyll content of maize under mulching treatments M1, M2, and M3 were 3.00%, 5.88% and 8.71% higher than M0 in monoculture, respectively. The planting pattern significantly affected the chlorophyll content of maize (*P<* 0.05). Under the four straw mulching levels from M0 to M3, the chlorophyll content of monoculture maize was 9.26%, 9.45%, 9.66%, and 10.2% higher than that of intercropped maize. The chlorophyll content of intercropped maize increased the most compared with monoculture maize under M3 treatment.

**Figure 7:**
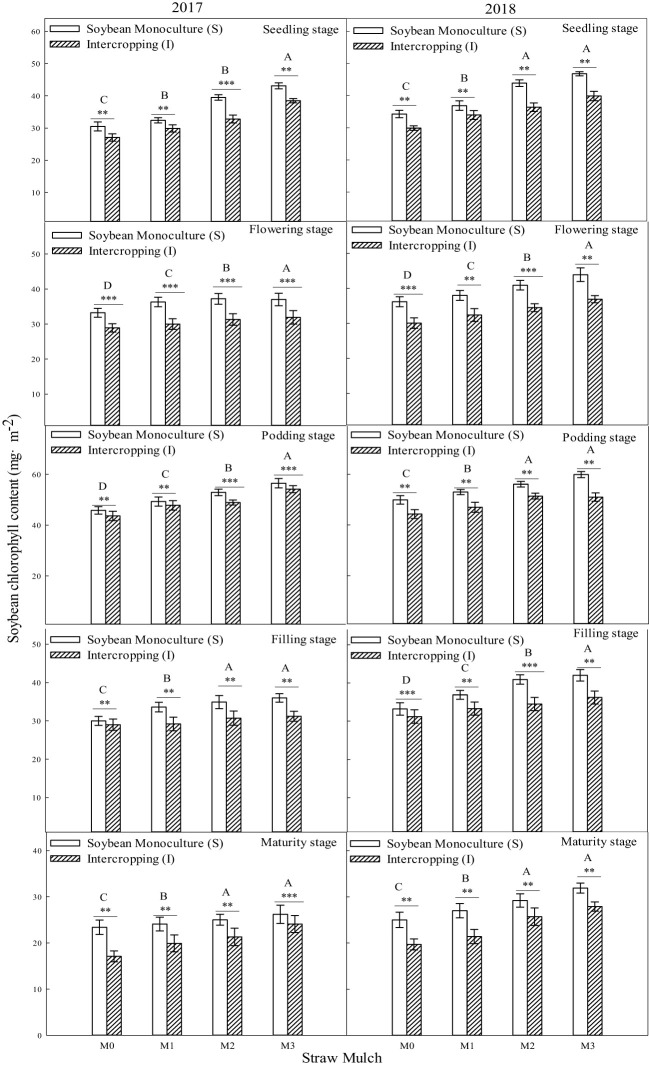
Effects of straw mulching and planting patterns on chlorophyll content of soybean at different growth stages. Bars labeled with different capital letters indicate significant differences between straw mulch treatments (*P<* 0.05). Straw mulching levels: 0 t ha^-1^ (M0), 4.8 t ha^-1^ (M1), 7.2 t ha^-1^ (M2), 9.6 t ha^-1^ (M3). ** and *** indicate significant differences at the *P* < 0.01, and *P* < 0.001 levels, respectively.

It can be seen from **Figure 7** that the chlorophyll content during the growth period of soybean was significantly affected by both straw mulching amount and planting pattern (*P<* 0.05). As the mulching amount increased from M0 to M3, the chlorophyl content of soybean consistently increased. Under intercropping, the chlorophyll content of soybean under mulching treatments M1 to M3 was 7.61%, 17.3%, and 24.0% higher than M0, respectively (based on two-year averages during the whole growth period). The chlorophyll content of soybean under mulching treatments M1, M2, and M3 was 7.19%, 14.7%, and 18.7% higher than M0 in monoculture. The planting pattern significantly affected the chlorophyll content of soybean (*P<* 0.05). Under the four straw mulching levels from M0 to M3, the chlorophyll content of monoculture soybean was 12.7%, 13.1%, 15.3%, and 17.7% higher than that of intercropped soybean. The chlorophyll content of intercropped soybean increased the most compared with that of monoculture soybean under M3 treatment.

### 3.4 Effects of straw mulching and intercropping on crop photosynthesis

**Figure 8:**
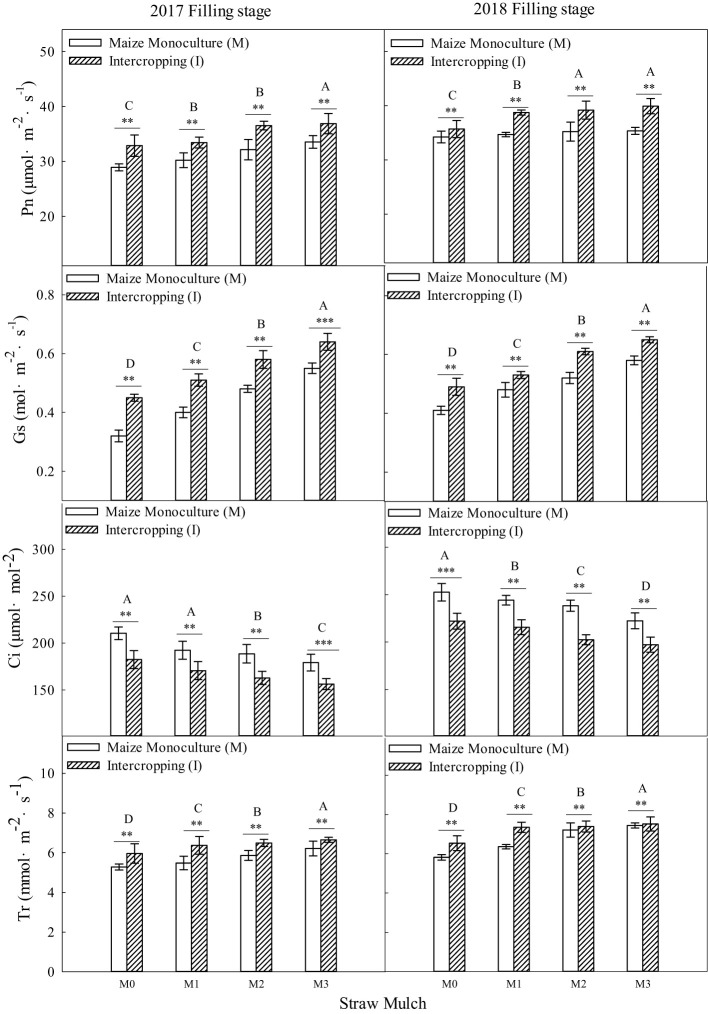
Effects of straw mulching and intercropping on maize photosynthesis at the filling stage. Bars labeled with different capital letters indicate significant differences in each photosynthetic parameter between straw mulch treatments (*P<* 0.05). Straw mulching levels: 0 t ha^-1^ (M0), 4.8 t ha^-1^ (M1), 7.2 t ha^-1^ (M2), 9.6 t ha^-1^ (M3). **, and *** indicate significant differences at the *P* < 0.01, and *P* < 0.001 levels, respectively. Pn, net photosynthetic rate; Sc, stomatal conductance; Tr, transpiration rate; Ci, intercellular carbon dioxide concentration.

**Figure 9:**
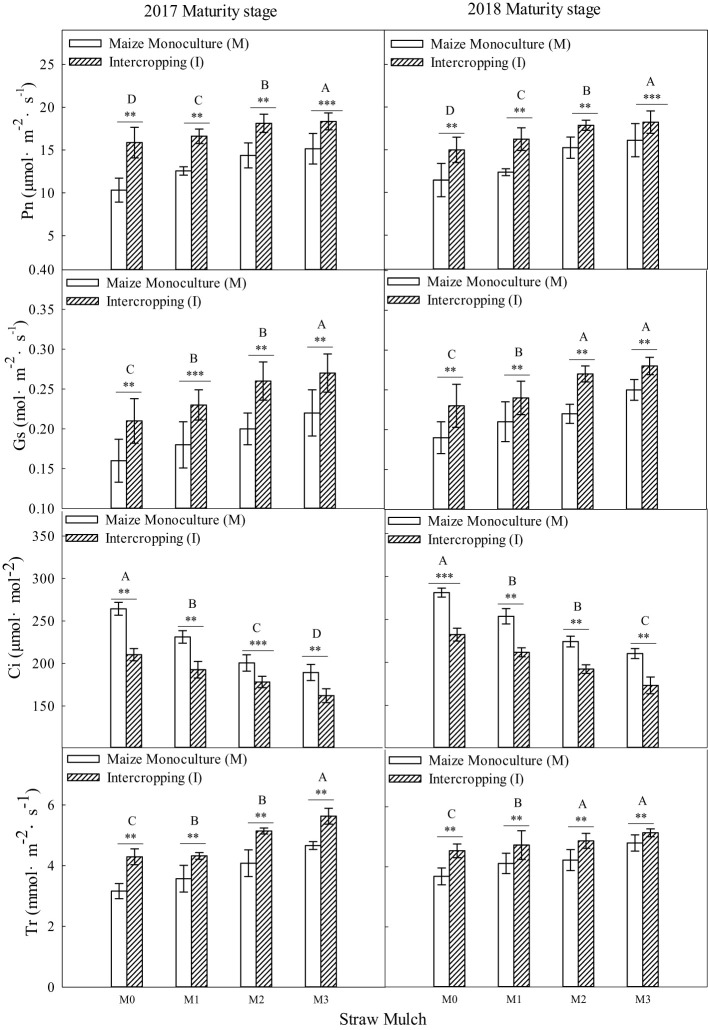
Effects of straw mulching and intercropping on maize photosynthesis at maturity. Bars labeled with different capital letters indicate significant differences in each photosynthetic parameter between straw mulch treatments (*P<* 0.05). Straw mulching levels: 0 t ha^-1^ (M0), 4.8 t ha^-1^ (M1), 7.2 t ha^-1^ (M2), 9.6 t ha^-1^ (M3). ** and *** indicate significant differences at the *P* < 0.01, and *P* < 0.001 levels, respectively. Pn, net photosynthetic rate; Sc, stomatal conductance; Tr, transpiration rate; Ci, intercellular carbon dioxide concentration.

As shown in **Figures 8**, **9**, straw mulching and planting patterns significantly affect maize photosynthetic parameters. The two years of results showed that intercropping significantly affected the photosynthetic characteristics of maize (*P<* 0.05).

Based on the average results from the two years of intercropped maize, under mulching treatments M1, M2, and M3. Pn was 3.64%, 11.3%, and 16.4% higher, Sc was 7.58%, 27.3%, and 43.9% higher, Tr was 14.8%, 27.6%, and 44.5% higher, and Ci was 10.9%, 18.7%, and 25.7% lower, respectively, relative to the M0 treatment. For maize monoculture, under mulching treatments M1, M2, and M3, Pn was 3.23%, 7.83%, and 11.4% higher, Sc was 6.72%, 22.7%, and 36.1% higher, Tr was 8.81%, 19.1%, and 28.8% higher, and Ci was 8.64%, 15.5%, and 20.6% lower, respectively, relative to the M0 treatment.

Straw mulching significantly affected the photosynthetic characteristics of maize (*P<* 0.05). Under the four straw mulching levels from M0 to M3 for intercropped maize, Pn was 10.6%, 11.0%, 14.1%, and 15.5% higher, Sc was 10.9%, 11.8%, 15.1%, and 17.3% higher, Tr was 10.5%, 16.6%, 18.3%, and 23.9% higher, and Ci was 12.0%, 14.2%, 15.3%, and 17.8% lower, respectively relative to monoculture maize.

**Figure 10:**
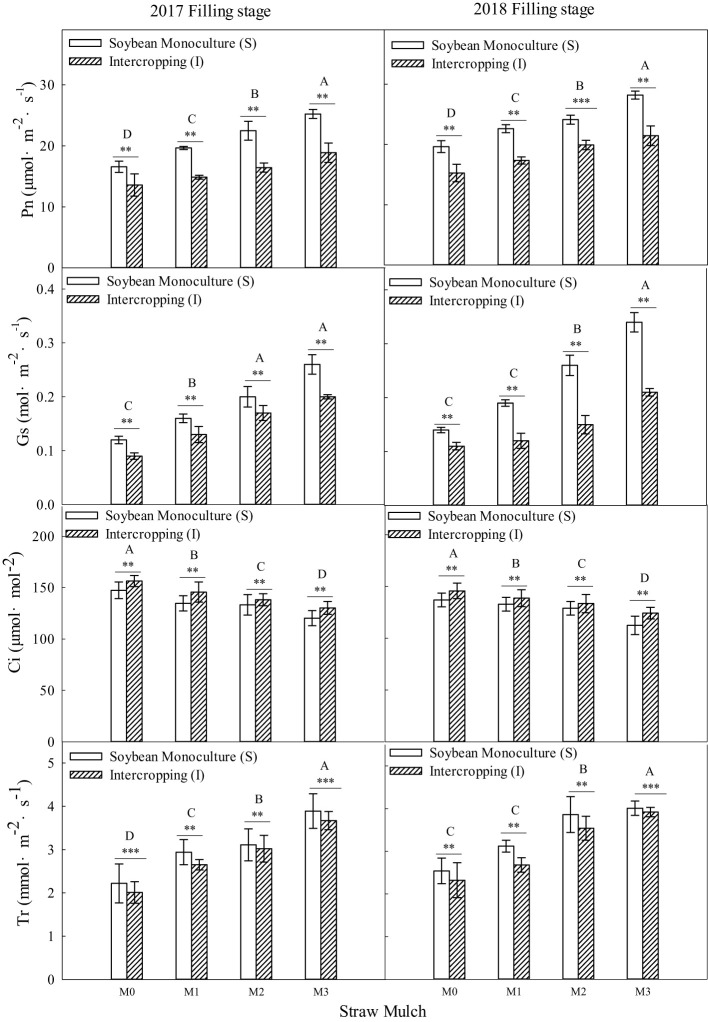
Effects of straw mulching and intercropping on soybean photosynthesis at the filling stage. Bars labeled with different capital letters indicate significant differences in each photosynthetic parameter between straw mulch treatments (*P<* 0.05). Straw mulching levels: 0 t ha^-1^ (M0), 4.8 t ha^-1^ (M1), 7.2 t ha^-1^ (M2), 9.6 t ha^-1^ (M3). ** and *** indicate significant differences at the *P* < 0.01, and *P* < 0.001 levels, respectively. Pn, net photosynthetic rate; Sc, stomatal conductance; Tr, transpiration rate; Ci, intercellular carbon dioxide concentration.

**Figure 11:**
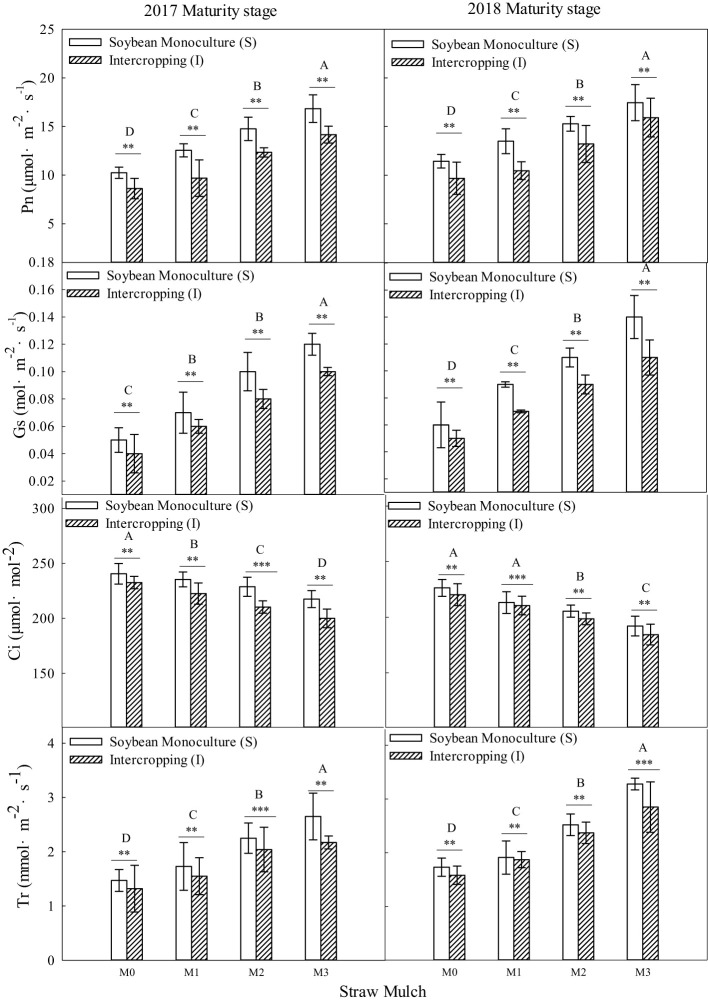
Effects of straw mulching and intercropping on soybean photosynthesis parameters at maturity. Bars labeled with different capital letters indicate significant differences in photosynthetic parameters between straw mulch treatments (*P<* 0.05). Straw mulching levels: 0 t ha^-1^ (M0), 4.8 t ha^-1^ (M1), 7.2 t ha^-1^ (M2), 9.6 t ha^-1^ (M3). ** and *** indicate significant differences at the *P* < 0.01, and *P* < 0.001 levels, respectively. Pn, net photosynthetic rate; Sc, stomatal conductance; Tr, transpiration rate; Ci, intercellular carbon dioxide concentration.

As shown in **Figures 10**, **11**, straw mulching and planting patterns significantly affected soybean photosynthetic parameters. The two years of results showed that intercropping had a significant effect on the photosynthetic characteristics of soybean (*P<* 0.05). For intercropped soybean, under mulching treatments M1, M2, and M3, Pn was 11.9%, 22.5%, and 35.0% higher, Sc was 7.96%, 15.9%, and 25.7% higher, Tr was 8.54%, 23.0%, and 35.1% higher, and Ci was 3.13%, 6.38%, and 10.8 lower, respectively relative to M0. For monoculture soybean, under these same mulching treatments, Pn was 18.0%, 32.4%, and 51.6% higher, Sc was 12.0%, 25.6%, and 41.9% higher, Tr was 9.17%, 26.0%, and 46.6% higher, and Ci was 6.73%, 12.4%, and 19.8% lower, respectively, relative to M0.

Straw mulching had a significant effect on the photosynthetic characteristics of soybean (*P<* 0.05). Under the four straw mulching levels from M0 to M3 in soybean monoculture, Pn was 11.0%, 17.0%, 19.9%, and 24.6% higher, Sc was 3.54%, 7.38%, 12.2%, and 16.9% higher, Tr was 8.66%, 9.29%, 11.3%, and 17.9% higher, and Ci was 4.14%, 7.71%, 10.3%, and 13.9% lower, respectively, relative to intercropped soybean.

### 3.5 Effects of straw mulching on nitrogen uptake in maize/soybean intercropping

**Figure 12:**
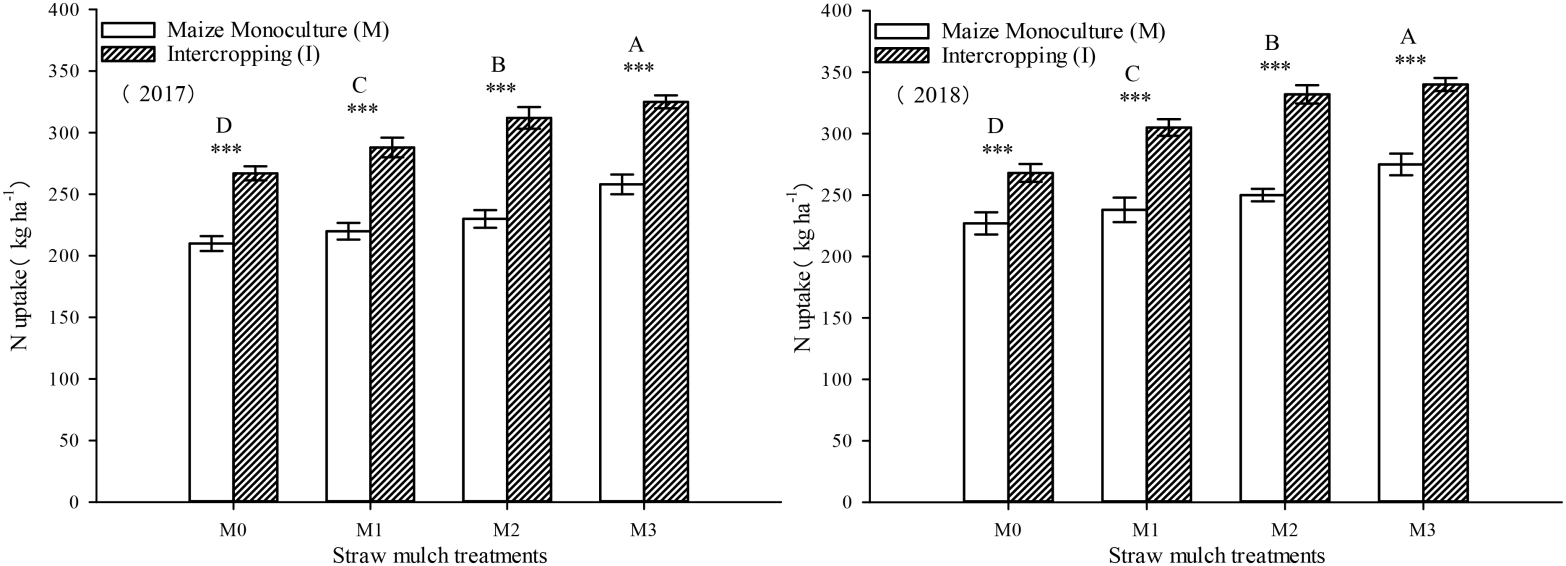
Effects of different straw mulching amounts on nitrogen uptake of maize in maize/soybean intercropping system in 2017 and 2018. Bars labeled with different capital letters indicate significant differences in nitrogen uptake between straw mulching treatments (*P<* 0.05). Straw mulching levels: 0 t ha^-1^ (M0), 4.8 t ha^-1^ (M1), 7.2 t ha^-1^ (M2), 9.6 t ha^-1^ (M3). *** indicate significant differences at the *P* < 0.001 levels, respectively.

This study investigated and analyzed the impact of straw mulching amount, planting pattern, and their interaction on the nitrogen uptake of maize. As shown in **Table 8**, all three factors had significant effects on the nitrogen uptake of maize (*P*< 0.05). Notably, the amount of straw mulching significantly impacted the nitrogen uptake of maize.Under intercropping, the nitrogen uptake of soybean under mulching treatments M1, M2, and M3 were 10.8%, 20.4%, and 24.3% higher than M0. In monoculture, the nitrogen uptake of soybean under these mulching treatments was 4.81%, 9.84%, and 22.0% higher than M0, respectively (**Figure 12**). Additionally, intercropping conditions positively influenced the nitrogen uptake of maize, leading to a significant increase of 22.4%, 29.5%, 34.2%, and 24.8% across all four straw mulching levels (M0 to M3, respectively) compared to monoculture maize over the two years. Notably, the highest nitrogen uptake was observed for intercropped maize under the M2 treatment.

**Table 8:** Analysis of variance of the effects of straw mulching amount and planting pattern on crop nitrogen uptake.

Source of variation	N					
	Maize		Soybean		Intercropping system	
	*F*	*P*	*F*	*P*	*F*	*P*
Planting pattern (P)	313.25	0.000	58.21	0.000	400.75	0.000
Straw mulch (M)	200.10	0.000	90.34	0.000	86.34	0.000
P×M	5.36	0.028	0.19	0.883	5.64	0.057

The values in the table represent the F- and P-values of the analysis of variance of the effects of straw mulching amount and planting pattern on crop nitrogen uptake. P, planting pattern; M, straw mulch.

**Figure 13:**
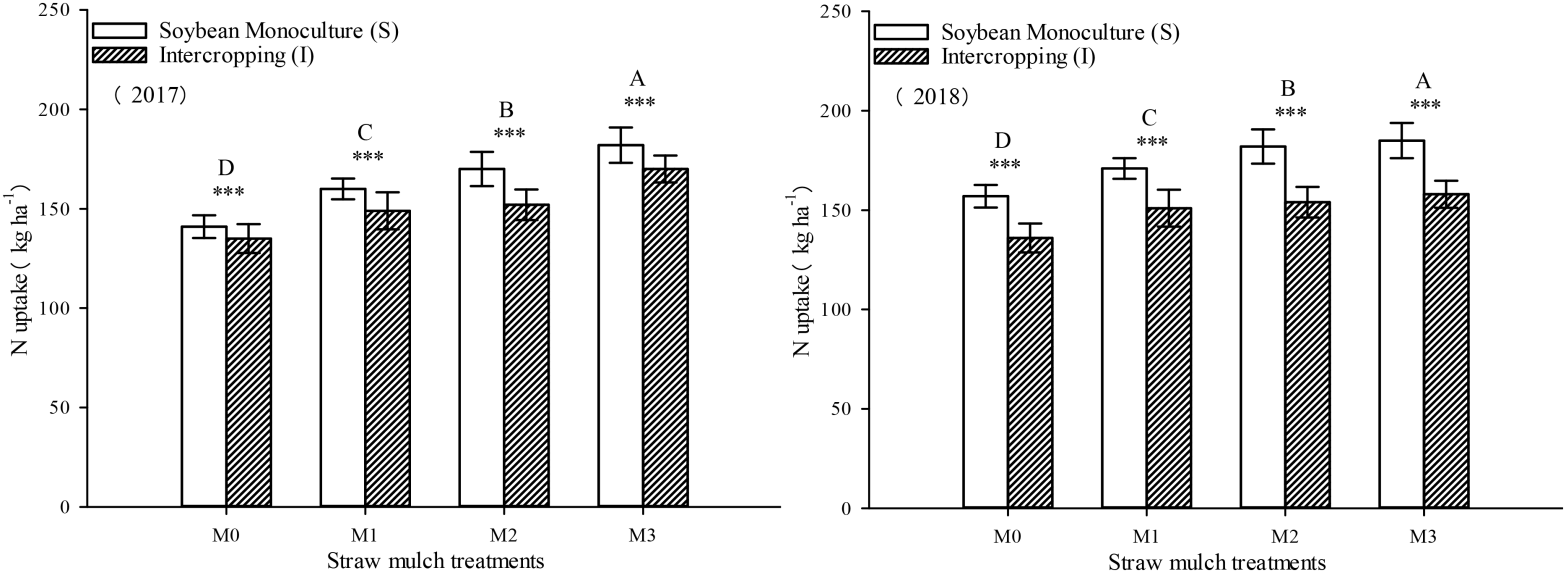
Effects of different straw mulching amounts on nitrogen uptake of soybean in maize/soybean intercropping system in 2017 and 2018. Bars labeled with different capital letters indicate significant differences in nitrogen uptake between straw mulch treatments (*P<* 0.05). Straw mulching levels: 0 t ha^-1^ (M0), 4.8 t ha^-1^ (M1), 7.2 t ha^-1^ (M2), 9.6 t ha^-1^ (M3). *** indicate significant differences at the *P* < 0.001 levels, respectively.

As shown in **Figure 13**, the nitrogen uptake of soybean was significantly affected by both straw mulching and planting patterns (*P<* 0.05). The amount of straw mulching significantly affected the nitrogen uptake of soybean plants, and the nitrogen uptake consistently increased with increased mulching. The nitrogen uptake of soybean under mulching treatments M1, M2, and M3 was 10.7%, 12.9%, and 21.0% higher, respectively, thanM0 in intercropping based on two-year averages. The nitrogen uptake of soybean under these mulching treatments was 11.1%, 18.1%, and 23.2% higher, respectively, thanM0, based on the two-year averages in monoculture. The planting pattern significantly affected the nitrogen uptake of soybean (*P<* 0.05). Under the four straw mulching levels from M0 to M3, the nitrogen uptake of monoculture soybean was 9.96%, 10.3%, 15.0%, and 11.9% higher than under intercropping based on two-year averages, respectively.

**Figure 14:**
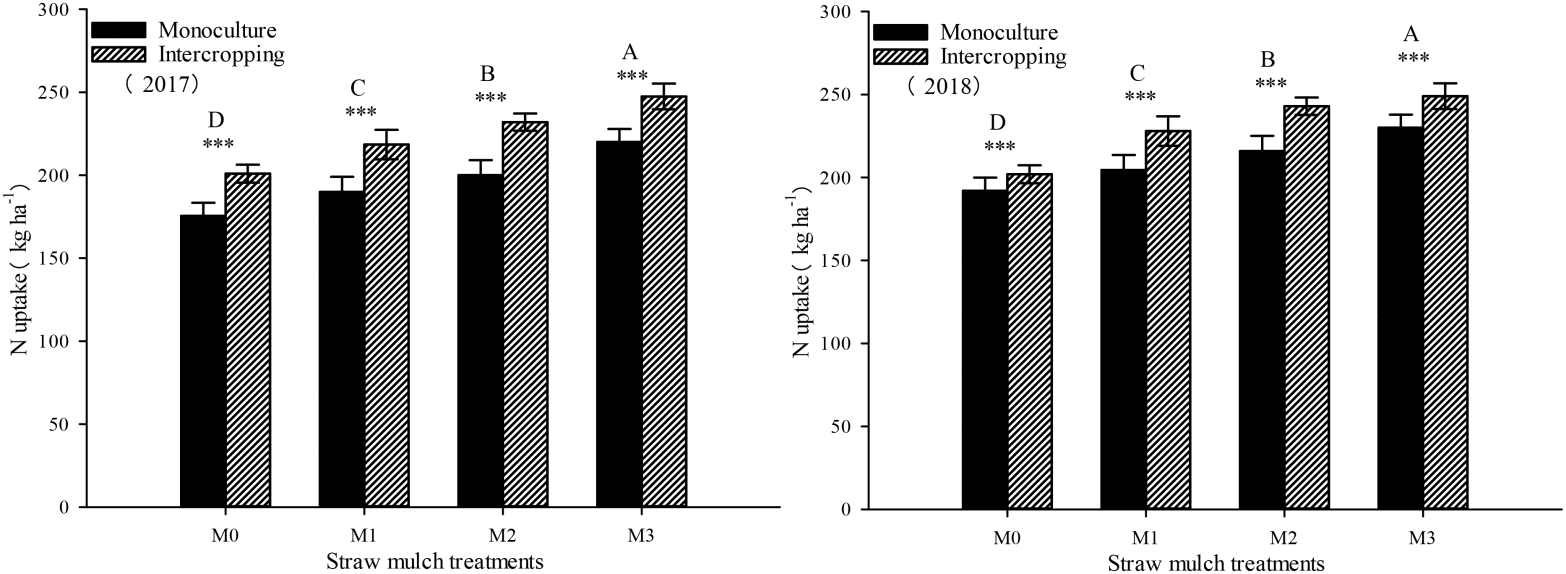
Effects of different straw mulching amounts on nitrogen uptake in the maize/soybean intercropping system in 2017 and 2018. Bars labeled with different capital letters indicate significant differences between straw mulch treatments (*P<* 0.05). Straw mulching levels: 0 t ha^-1^ (M0), 4.8 t ha^-1^ (M1), 7.2 t ha^-1^ (M2), 9.6 t ha^-1^ (M3). *** indicate significant differences at the *P* < 0.001 levels, respectively.

As seen in **Figure 14**, the total nitrogen uptake of the entire intercropping system consistently increased with thestraw mulching amount, reaching its maximum value under M3 treatment, which was significantly higher than that of the other three treatments. Under the four straw mulching treatments M0–M3, intercropping significantly increased nitrogen uptake by 9.66%, 13.2%, 14.2%, and 10.3%, respectively, compared with monoculture based on two-year averages. Under M2 treatment, the difference between monoculture and intercropping was the largest. As seen in **Table 8**, the straw mulching amount and planting pattern had a significant effect on the total nitrogen absorption of the system (*P<* 0.05).

### 3.6 Effects of straw mulching and intercropping on crop yield and yield-related traits

**Figure 15:**
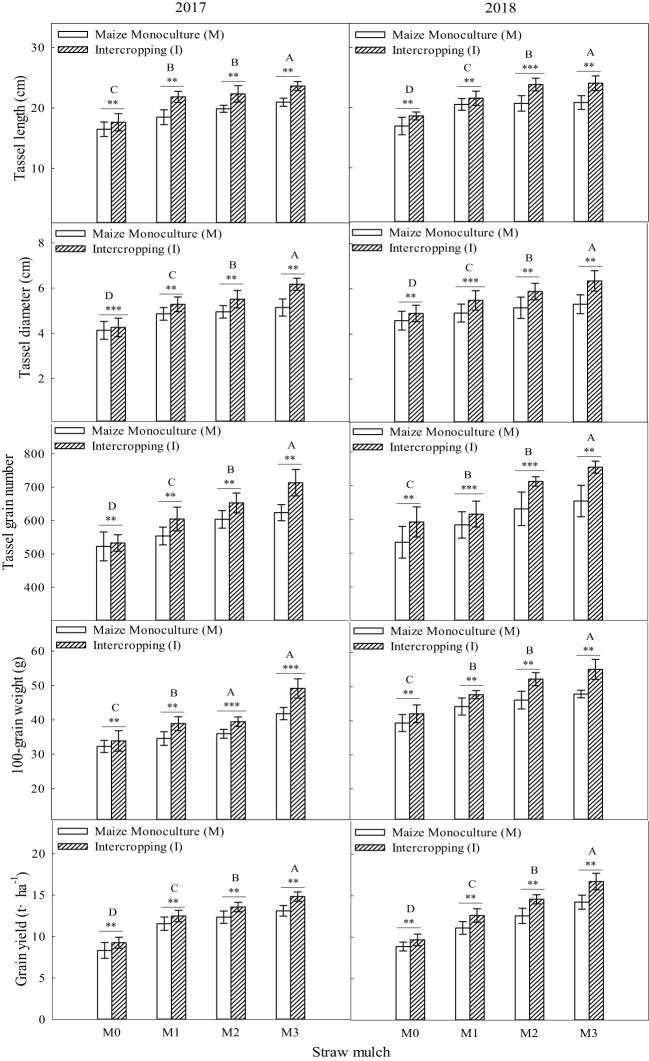
Effects of straw mulching and intercropping on maize yield and yield-related traits. Bars labeled with different capital letters indicate significant differences between straw mulch treatments (*P<* 0.05). Straw mulching levels: 0 t ha^-1^ (M0), 4.8 t ha^-1^ (M1), 7.2 t ha^-1^ (M2), 9.6 t ha^-1^ (M3). ** and *** indicate significant differences at the *P* < 0.01, and *P* < 0.001 levels, respectively.

As shown in **Figure 15**, maize yield and its components were significantly affected by straw mulching and planting patterns (*P<* 0.05). Under the four straw mulching levels, the yield components of intercropped maize were significantly higher than those of monoculture maize (*P<* 0.05). For intercropped maize, under the four straw mulching levels from M0 to M3, tassel length was 8.50%, 11.2%, 13.8%, and 14.1% higher, tassel diameter was 5.16%, 10.0%, 12.7%, and 19.7% higher, tassel grain number was 6.65%, 7.27%, 10.6%, and 14.90% higher, 100-grain weight was 6.02%, 9.93%, 11.8%, and 16.3% higher, and yield was 10.1%, 10.7%, 13.0% and 15.4% higher, respectively, than that of monoculture maize based on two-year averages.

Straw mulching significantly affected maize yield and its components (*P<* 0.05). Based on the results of the two years, the tassel length, tassel diameter, tassel grain number, 100-grain weight, and yield of maize each consistently increased with the increase of straw mulching under intercropping and monoculture.

**Figure 16:**
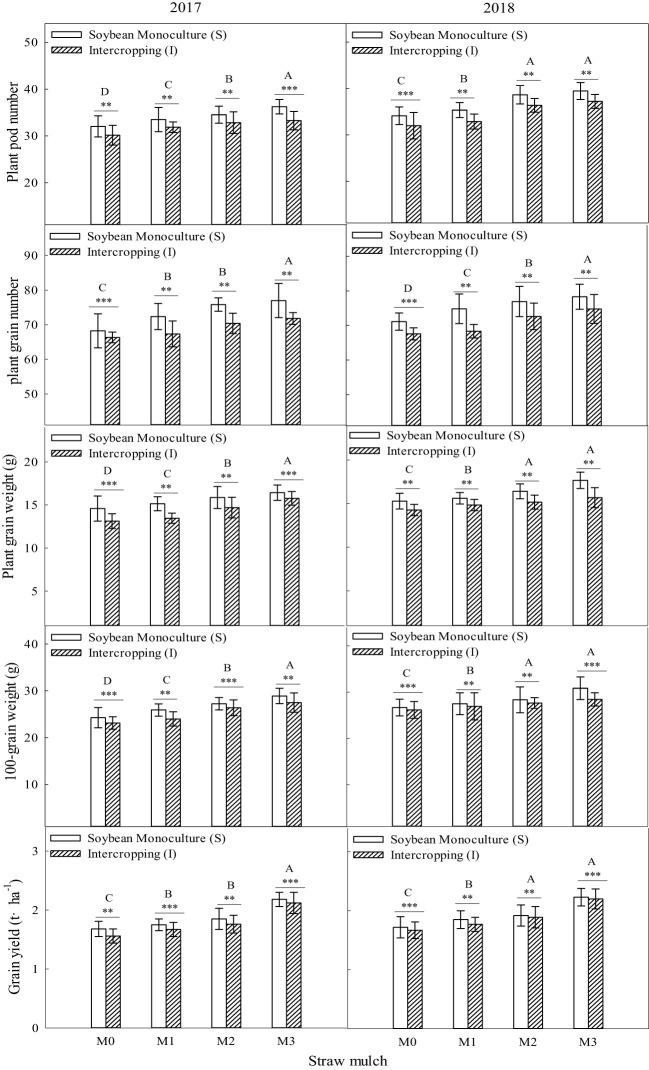
Effects of straw mulching and intercropping on soybean yield and yield-related traits. Bars labeled with different capital letters indicate significant differences between straw mulch treatments (*P<* 0.05). Straw mulching levels: 0 t ha^-1^ (M0), 4.8 t ha^-1^ (M1), 7.2 t ha^-1^ (M2), 9.6 t ha^-1^ (M3). ** and *** indicate significant differences at the *P* < 0.01, and *P* < 0.001 levels, respectively.

It can be seen from **Figure 16** that soybean yield and its components were significantly affected by straw mulching and planting patterns (*P<* 0.05). Under the four straw mulching levels, the yield components of monoculture soybean were considerably higher than those of intercropped soybean (*P<* 0.05). For monoculture soybean, under the four straw mulching levels from M0 to M3, per plant pod number was 6.45%, 8.50%, 10.1%, and 15.9% higher, per plant grain number was 4.05%, 8.48%, 9.09%, and 10.2% higher, per plant grain weight was 9.06%, 9.59%, 11.5%, and 12.8% higher, 100-grain weight was 3.37%, 8.84%, 9.59%, and 15.8% higher, and yield was 5.26%, 6.19%, 8.77%, and 9.03%higher, respectively, than that of intercropped soybean based on two-year averages.

Straw mulching significantly affected soybean yield and its components (*P<* 0.05). Based on the results of the two years, per plant pod number, per plant grain number, per plant grain weight, 100-grain weight, and yield of soybean increasedconsistently with the straw mulching amount under both intercropping and monoculture.

## 4 Discussion

The present two-year field study in Northeast China showed that intercropping maize and soybean with straw mulching improved the growth performance and grain yield of both crops (maize and soybean) compared withno mulching. Moreover, the performance of maize was improved by intercropping relative to monoculture conditions. This is consistent with previous studies investigating intercropping of various crops and finding better combined growth indices and yields of cereals and legumes. Many studies have shown intercropping advantages in different intercropping systems (Li et al., 2009; Fang et al., 2010). Intercropping often results in better crop performance and grain yield relative to monocropping systems by utilizing available resources moreeffectively (Raza et al., 2021). Maize/soybean intercropping enhanced the capture and utilization ofresources overall compared with mono-croppingowing to the complementary resource use of both crops in the intercrop system (Liu et al., 2018; Li C. et al., 2020). One crop can improve the growth and development of another crop and increase the activity of individual plants through the reciprocity between species in the intercropping systemsuch that the economic benefits of the intercropping systemare higher than that of crop monocultures (Hauggaard-Nielsen and Jensen, 2005).

A global analysis of the many advantages of intercropping showed that the land equivalent ratio of intercropping worldwide is around 1.3 (Martin et al., 2018). A maize/soybean intercropping system increases the total energy output value by 38% (Martin et al., 2018), and the land equivalent ratios of oat/maize intercropping, oat/sunflower intercropping, and oat/mung bean intercropping have been estimated at 1.10–1.4, 1.23–1.38, and 1.05–1.08, respectively (Qian et al., 2018). Intercropping has advantages in fertilizer and land use, mainly because intercropping concentrates production on less land under the same nitrogen fertilizer input per unit area. The present study found that maize intercropping had a noticeable advantage compared with monoculture. Under the same straw mulching level, the plant height (**Figure 2**), stem diameter (**Figures 4**, **5**), andLAI (**Table 7**) of intercropped maize were significantly higher than those of monoculture maize (*P<* 0.05). The improved growth of both crops in the intercropping system (measured as increased leaf area index and dry matter accumulation) is most likely associated with higher water use efficiency, improved light use efficiency, and nutrient accumulation (Rahman et al., 2017; Ahmed et al., 2018; Liu et al., 2018). Moreover, efficient uptake and utilization of nutrients and soil waterenhance root system proliferation and lead to improved crop growth by promoting crop plant height, stem diameter, leaf area, and other growth indicators (Mao et al., 2012; Chen et al., 2017; Li et al., 2020). Soybean in a maize/soybean intercropping system exhibits an inhibition of growth, so the intercropped soybean plant height (**Figure 3**), stem diameter (**Figure 5**), and leaf area index (**Table 6**) were significantly less than those of monoculture soybean (*P<* 0.05).

Straw mulching can promote crop growth and development, increase leaf area index and leaf chlorophyll content, and improve aboveground dry matter quality (Qin et al., 2010; Khan et al., 2022a). The leaf area index is a positive indicatorof improved yield and lower evaporation and is critical to maintaining higher intercropping yield (Kamara et al., 2017). Studies in soybean have shown that straw mulching can enhance the growth and development of plants, increase nodule weight, nodule number, LAI, and chlorophyll content, increase growth indexes (such as plant height and stem diameter), and, ultimately, increase the biomass and economic yield (Sekhon et al., 2005; Khan et al., 2022a). The present study showed that maize straw mulching could increase plant height (**Figures 2**, **3**), stem diameter (**Figures 4**, **5**), LAI (**Tables 7**, **6**), and chlorophyll content (**Figures 6**, **7**) of rain-fed maize and soybean crops, supporting previous research results (Khan et al., 2022a).

Two years of experimental data have shown that intercropping has a positive effect on the photosynthetic performance of crops (**Figures 8**–**11**). Additionally, intercropping of tall and short plants creates an umbrella canopy structure during the overlapping growth period, which increases the incident radiation on both sides of crop rows and allows for full exposure of the leaves in the middle of the canopy to light, thus promoting photosynthesis (Lin et al., 2020). The improved photosynthetic characteristics of maize in intercropping are also owing to the ventilation and light conditions being optimized by intercropping soybean, which is a short crop, with maize, a tall crop (Xiangqian, et al., 2014). This increases the photosynthetic performance parameters of maize in the maize/soybean intercropping system, while simultaneously weakening the photosynthetic performance parameters of soybean (**Figures 8**–**11**). Furthermore, straw mulchingwas also found to improve the photosynthetic characteristics of maize and soybean. The level of improvement increased with increasing levels of mulching, with the improvement mainlyconsistent with enhanced water use, growth, and development of maize (Liu et al., 2019) (**Figures 8**–**11**). Overall, these findings suggest that intercropping and straw mulching have the potential to significantly improve crop productivity and sustainability, thereby contributing to sustainable food production.

It has been shown that straw mulching can improve the net photosynthetic rate (Pn) of functional leaves of winter wheat (Zhai et al., 2021). In addition, studies have found that the effects of straw mulching on crop photosynthetic parameters, including Pn, Sc, Tr, and Ci, are indirect (Liu et al., 2019). Mulching improves the soil environment, increases the absorption and utilization of nutrients and water by crops, and promotes the growth of roots (He et al., 2017), thereby increasing the morphological indicators of crops, such as leaf area, and internal physiological indicators, which ultimately improves the light interception and growth of crops (Retta et al., 2016). The present study also confirmed that under mulching, the nitrogen uptake of maize and soybean crops increased with the increase of straw mulching (**Figures 12**–**14**), which would also explain the ultimate rise in crop yield (Yu et al., 2010). Intercropping has obvious yield advantages, whichare based on the effective use of nutrient resources (Willey, 1979; Wang Z. et al., 2023), and is the result of the combined effect of nutrient uptake and utilization efficiency of intercropped crops (Chowdhury and Rosario, 1994).Regarding the yield-increasing effect of straw mulching, although there are some differences in the yield results of each test treatmentowing to different study regions, years, and mulching amounts, overall, within a given coverage range, crop yield increases withincreasing coverage. Inthe present study, a crop yield advantage in the intercropping system was found under all straw mulching levels. Straw mulching significantly increased crop yield (**Figures 15**, **16**), which is similar to the results of previous studies (Khan et al., 2021; Runzhi et al., 2021; Du et al., 2022). Crop yield and dry matter quality are closely related to crop photosynthetic characteristics. Under straw mulching, crop photosynthetic performance is improved, and as crop yield and dry matter quality are the accumulation of photosynthetic products, they also increase (Monti et al., 2005).

## 5 Conclusions

The two years of results showed that straw mulching and intercropping can promote the growth of crops, significantly increase the plant height and stem diameter of crops, increase the leaf area and chlorophyll content of crops, and thus promote the photosynthesis of crops, increase the nitrogen absorption of crops, and ultimately increase the yield of crops. The strongest effect was observed under a coverage of 9.6 t ha^-1^ maize straw under maize/soybean intercropping. Thus, in the actual agricultural production process, agricultural measures combining straw mulching and crop intercropping can be adopted to improve the leaf traits and photosynthetic physiological characteristics of crops, so as to achieve the goal of optimizing crop growth and development status while increasing crop yield.

## Data availability statement

The original contributions presented in the study are included in the article/supplementary material. Further inquiries can be directed to the corresponding authors.

## Author contributions

SL: Conceptualization, Data curation, Formal Analysis, Methodology, Software, Writing – original draft. IK: Writing – review & editing. FN: Data curation, Visualization, Writing – review & editing. AR: Conceptualization, Data curation, Writing – review & editing. RS: Conceptualization, Writing – review & editing. LC: Conceptualization, Data curation, Writing – review & editing. LW: Funding acquisition, Investigation, Project administration, Resources, Supervision, Validation, Writing – original draft.
